# Melatonin Treatment for Sleep Disorders in Parkinson's Disease: A Meta-Analysis and Systematic Review

**DOI:** 10.3389/fnagi.2022.784314

**Published:** 2022-02-04

**Authors:** Hongxia Ma, Junqiang Yan, Wenjie Sun, Menghan Jiang, Yongjiang Zhang

**Affiliations:** ^1^Key Laboratory of Neuromolecular Biology, The First Affiliated Hospital, College of Clinical Medicine of Henan University of Science and Technology, Luoyang, China; ^2^Department of Neurology, The First Affiliated Hospital, College of Clinical Medicine of Henan University of Science and Technology, Luoyang, China

**Keywords:** melatonin, Parkinson's disease, sleep disorders, meta-analysis, systematic review

## Abstract

**Objective:**

The efficacy of melatonin on sleep disorders in Parkinson's disease (PD) is still unclear. The purpose of this study was to investigate the efficacy of melatonin on sleep disorders in PD by summarizing evidence from randomized clinical trials (RCTs).

**Methods:**

PubMed, Cochrane Library, EMBASE, and Web of Science databases were searched for studies published before 20 August 2021. Results were analyzed using Review Manager 5.2 software. We used Trial Sequential Analysis (TSA) software to avoid false-positive results caused by random errors.

**Results:**

We included 7 studies in this systematic review and meta-analysis. The results of the meta-analysis showed that compared with placebo, the subjective sleep quality of patients with PD significantly improved after melatonin treatment (MD = −2.19, 95% CI: −3.53 to −0.86, *P* = 0.001). In the systematic review, we qualitatively analyzed the efficacy of melatonin on the objective sleep quality of patients with PD, and the results showed that melatonin exerted a positive effect with good safety and tolerability. However, there was no significant improvement in excessive daytime sleepiness assessed by the Epworth Sleepiness Scale (ESS).

**Conclusion:**

We found that melatonin can significantly improve the subjective and objective sleep quality of patients with PD with good safety and tolerability. Melatonin could be considered an effective treatment for insomnia in patients with PD.

## Introduction

Parkinson's disease (PD), the second most common progressive neurodegenerative disorder, is characterized by motor and non-motor symptoms. The prevalence of PD increases with age (Lang and Lozano, 1998a,b; Tysnes and Storstein, 2017; Hayes, 2019). The Global Burden of Disease Study showed that the number of PD cases worldwide will double from approximately 7 million in 2015 to approximately 13 million in 2040, which may bring a tremendous burden to society (GBD 2016 Parkinson's Disease Collaborators, 2018).

Sleep disorders are a common non-motor symptom in patients with PD (Schrempf et al., 2014). The up 88% to 98% of patients with PD have sleep disorders, such as insomnia, daytime sleepiness with sleep attacks, and rapid eye movement sleep behavior disorder (RBD), which has an enormous negative impact on the quality of life of patients with PD (Schrempf et al., 2014; Ahn et al., 2020). Patients with PD had blunted circadian rhythms of melatonin secretion, and both the amplitude of the melatonin rhythm and the 24-h area-under-the-curve for circulating melatonin levels were significantly lower in PD participants, which indicated that circadian dysfunction may underlie excessive daytime sleepiness in PD (Videnovic et al., 2014).

Melatonin, a neurohormone, is synthesized and secreted by the pineal gland at night (Palagini et al., 2021). Melatonin has chronobiotic action, which can entrain the circadian rhythms of several biological functions, including sleep/wake rhythms, and it also has sleep-promoting action (McCall et al., 2017; Geoffroy et al., 2019). Melatonin has positive effects on sleep quality in adults with respiratory diseases, metabolic disorders, and primary sleep disorders (Fatemeh et al., 2021). However, the efficacy of melatonin on sleep disorders in patients with PD is still unclear. Lyashenko et al. concluded that melatonin can be used for RBD treatment in patients with PD (Lyashenko et al., 2015). However, the latest randomized controlled trials showed that melatonin was ineffective for PD-RBD (Ahn et al., 2020; Gilat et al., 2020). Although Zhang et al. performed a meta-analysis to evaluate the effect of exogenous melatonin on sleep disorders in neurodegenerative diseases (Zhang et al., 2016), they did not systematically evaluate the effect of melatonin on other sleep disorders in patients with PD, such as excessive daytime sleepiness. Due to the inconsistency and incompleteness of the existing research results, we planned to perform a meta-analysis of RCTs and a systematic review to comprehensively investigate the efficacy of melatonin in the treatment of sleep disorders in patients with PD, such as subjective and objective sleep quality, excessive daytime sleepiness, and PD-RBD.

## Methods

### Search Strategy

Studies published before 20 August 2021 were searched in PubMed, Cochrane Library, EMBASE, and Web of Science. The keywords were entered using a standard search and included “Parkinson's disease,” “melatonin,” and “sleep disorders”. There was no restriction on language or date. Two authors screened the titles and abstracts independently. This protocol was conducted following the Preferred Reporting Items guidelines for Systematic Reviews and Meta-analyses (PRISMA) Protocols. We used PubMed as an example in the detailed search strategy presented in Supplementary Material 1.

### Inclusion and Exclusion Criteria

The inclusion criteria were as follows: (1) Study type: randomized controlled trials (RCTs); (2) Participants: patients who were clinically diagnosed with PD; (3) Interventions: the experimental group was given melatonin or prolonged-release melatonin (PRM); (4) Control: the control group was given placebo or clonazepam; and (5) Outcome: at least one of the following 4 instruments was employed: Pittsburgh Sleep Quality Index (PSQI), Epworth Sleepiness Scale (ESS), RBD questionnaire (RBDQ) and polysomnography (PSG) sleep parameters.

Exclusion criteria were: (1) Non-randomized controlled trial; (2) Repeated publication; and (3) Studies whose outcomes did not meet our meta-analysis requirements.

### Data Extraction

The following data were collected from each included study: (1) baseline characteristics including the first author's name, year of publication, country, study design, age, sample size, sex ratio, duration of treatment, measuring tools, daily dose of melatonin supplementation, and duration of PD; (2) mean and standard deviation (SD) of PSQI scores at the end of treatment. Two researchers extracted the required data for this meta-analysis and systematic review. The third independent researcher resolved disagreements and differences, if necessary.

### Assessment of Risk of Bias

The risk of bias of each RCT was assessed independently by two authors, and another author resolved any disagreement. We used the Cochrane Risk of Bias Tool to assess the risk of bias. The assessment tool is composed of seven parts: (1) random sequence generation; (2) allocation consultation; (3) blinding of the participants and personnel; (4) blinding of outcome assessment; (5) incomplete outcome data; (6) selective reporting; and (7) other bias. We divided the research into three categories, including “low risk of bias,” “high risk of bias,” or “unclear risk of bias”.

### Statistical Analysis

We used Review Manager (RevMan 5.2) to conduct this meta-analysis. We calculated the mean difference (MD) among the continuous variable data. The 95% confidence interval (CI) was used to represent each effect size. The *p*-value of < 0.05 was considered to indicate statistical significance. We estimated the heterogeneity between studies using I^2^ statistics. When *I*^2^ < 50%, there was no significant heterogeneity in the included studies, and a fixed-effects model was applied. When *I*^2^ ≥ 50%, there was heterogeneity, and a random-effects model was applied. To avoid false-positive results caused by random errors, we conducted the analysis using Trial Sequential Analysis (Copenhagen Trial Unit, Centre for Clinical Intervention Research, Rigshospitalet, Copenhagen, Denmark, https://www.ctu.dk/tsa).

## Results

### Selected Studies and Characteristics

**Figure 1:**
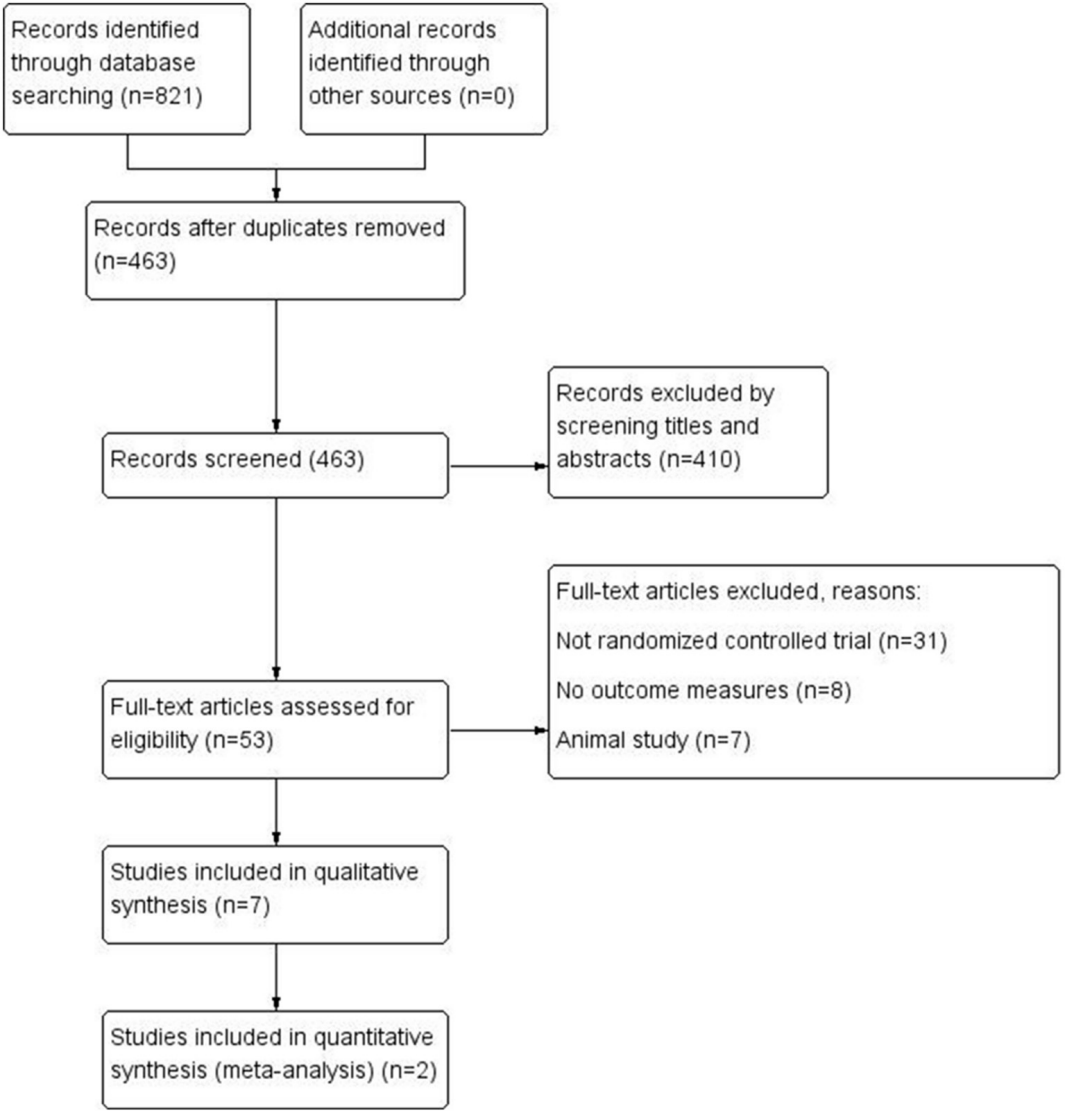
Study flow diagram.

As shown in Figure 1, we identified 821 records from the PubMed, Cochrane Library, EMBASE, and Web of Science databases. After excluding duplicates and irrelevant studies by reading titles and abstracts, the remaining 53 articles required reading of the full text to identify available data. Forty-six articles were excluded. Finally, we included 7 studies in this systematic review and meta-analysis. The characteristics of the included studies are shown in Table 1.

**Table 1:** Basic characteristics of included studies.

**References**	**Country**	**Study design**	**Intervention**	**Control**	**Sample size**	**Mean age (years) intervention**	**Control**	**Gender (M/F) intervention**	**Control**	**Duration (weeks)**	**Daily dose (mg)**	**Measuring tools**	**Duration Of PD (years) intervention**
Daneshvar Kakhaki et al. (2020)	Iran	Parallel	Melatonin	Placebo	51	64.4	66.3	16/9	16/10	12	10	PSQI scale	5.7
Ahn et al. (2020)	South Korea	Parallel	PRM	Placebo	34	66	64.6	8/8	9/9	4	2	PSQI scale RBDQ ESS scale	5.0
Dowling et al. (2005)	US	Cross-over	Melatonin	Placebo	40	61.7	61.7	29/11	29/11	2	5	ESS scale	7.5
Gilat et al. (2020)	Australia	Parallel	PRM	Placebo	30	-	-	-	-	12	4	RBDQ	-
Medeiros et al. (2007)	Brazil	Parallel	Melatonin	Placebo	18	62.9	60.7	7/1	7/3	4	3	PSQI scale ESS scale PSG	6.4
Litvinenko et al. (2012)	Russia	Parallel	Melatonin	Clonazepam	38	-	-	-	-	6	3	ESS scale PSG	-
Delgado-Lara et al. (2020)	Mexico	Cross-over	Melatonin	Placebo	26	52.5	57	7/5	10/4	12	50	ESS	3.5

*PRM, prolonged release melatonin; PSQI, Pittsburgh sleep quality index; ESS, Epworth Sleepiness Scale; RBDQ, RBD questionnaire; PSG, Polysomnography*.

### Risk of Bias Assessment

**Figure 2:**
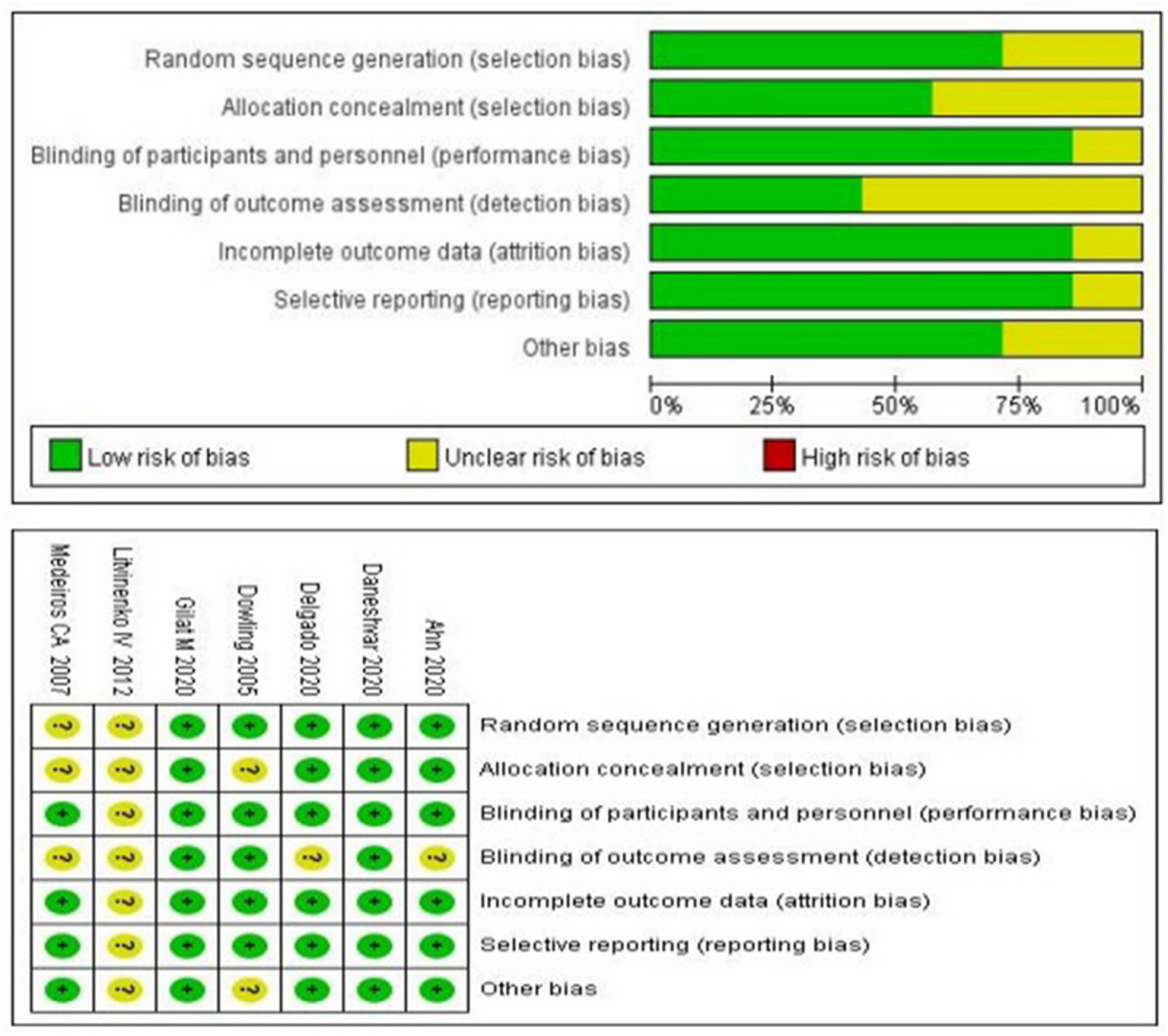
Quality assessment of the included studies.

The Cochrane Risk of Bias Tool was used to assess the risk of bias. The results are shown in Figure 2.

### Effect of Melatonin on Insomnia

Three studies (Medeiros et al., 2007; Ahn et al., 2020; Daneshvar Kakhaki et al., 2020) used the PSQI scale to assess the subjective sleep quality of patients with PD. However, only two studies (Medeiros et al., 2007; Daneshvar Kakhaki et al., 2020) reported the mean and SD of PSQI scores at the end of treatment. One study (4-week, randomized, double-blind, placebo-controlled) showed that compared with baseline and the placebo group, the PSQI score significantly improved in patients with PD treated with 4 weeks of 2 mg prolonged-release melatonin (PRM) (Ahn et al., 2020). These results showed that in the PRM group, the mean change in the PSQI score at the end of treatment was 1.75 (18.4%) (95% CI: 0.33–3.17; *p* = 0.049; Ahn et al., 2020). We also found that in the PRM group, the PSQI subcomponents improved, including subjective sleep quality (mean difference = 0.38; *p* = 0.029), sleep latency (mean difference = 0.38; *p* = 0.029), and sleep disturbance (mean difference = 0.25; *p* = 0.041; Ahn et al., 2020). However, in the Ahn et al. study, the mean and SD of PSQI scores after treatment could not be extracted. Therefore, we performed a qualitative analysis in our study.

**Figure 3:**
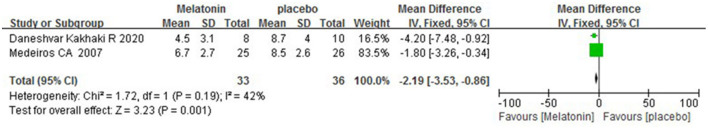
Forest plot of the efficacy of melatonin on the subjective sleep quality of patients with PD.

Two studies (Medeiros et al., 2007; Daneshvar Kakhaki et al., 2020) reported the mean and SD of PSQI scores. Our meta-analysis results showed that compared with the placebo group, the subjective sleep quality of patients with PD had a significant improvement after receiving melatonin treatment (2 studies, *n* = 69, MD = −2.19, 95% CI: −3.53 to −0.86, *P* = 0.001; Heterogeneity: Chi^2^ = 1.72, *I*^2^ = 42%, *P* = 0.19; see Figure 3). A fixed-effects model was applied.

**Figure 4:**
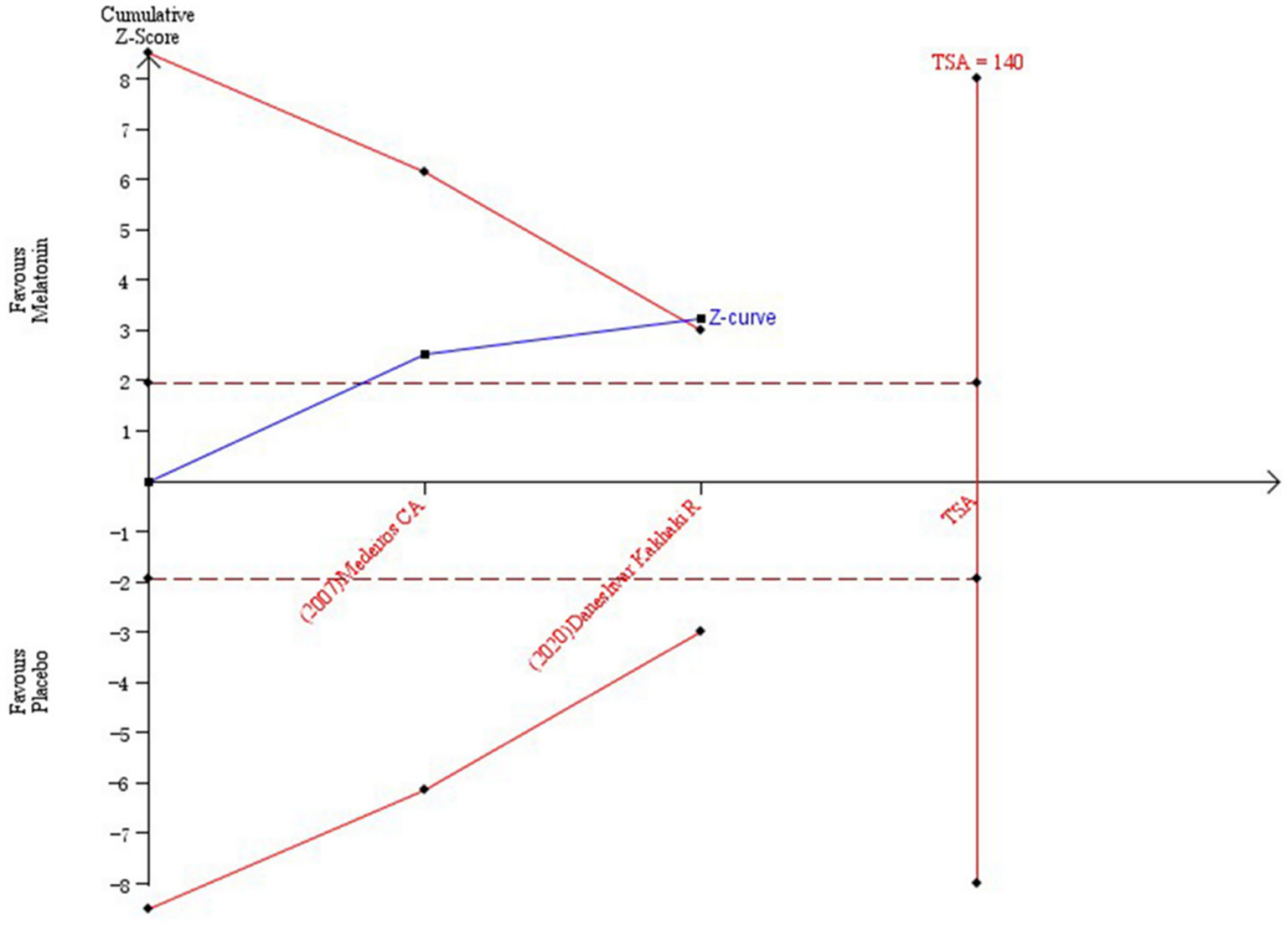
Trial sequential analysis of the cumulative meta-analysis of the efficacy of melatonin vs. placebo on subjective sleep quality in patients with PD.

The results of TSA on the data of PSQI scores at the end of treatment are shown in Figure 4. The accumulated Z value of the meta-analysis crossed both the TSA boundary value and the traditional boundary value before the required information size of 140 was reached.

Two studies (Medeiros et al., 2007; Litvinenko et al., 2012) used PSG sleep parameters to assess the objective sleep quality of patients with PD after melatonin treatment. In a randomized, double-blind, parallel-group, placebo-controlled study, the authors did not observe a significant difference in polysomnographic measures after treatment with 4 weeks of 3 mg melatonin. However, total sleep time (TST) improved in the melatonin-treated group (Medeiros et al., 2007). The sample size and a type II error may be causes of the undetected differences in PSG findings. In another study, Litvinenko et al. found that changes in PSG scores at the end of week 6, compared with the beginning of the trial, were in favor of the group treated with melatonin (Litvinenko et al., 2012). Their results showed that significant changes in total sleep time/time in bed (TST/TIB) and sleep latency (SL) were observed after treatment with 6 weeks of 3 mg melatonin (*p* = 0.001, *p* = 0.004, respectively).

### Effect of Melatonin on Daytime Sleepiness

Five studies (Dowling et al., 2005; Medeiros et al., 2007; Litvinenko et al., 2012; Ahn et al., 2020; Delgado-Lara et al., 2020) used the Epworth Sleepiness Scale to assess the daytime sleepiness of patients with PD. The study of Medeiros showed that in the melatonin group, the mean change in ESS score at the end of treatment was 0.3; it was 0.2 in the placebo group. However, the difference was not statistically significant. The trial showed that treatment with 4 weeks of 3 mg melatonin did not affect daytime sleepiness in patients with PD. In a 6-week, randomized, clonazepam-controlled study, Litvinenko et al. reported that melatonin and clonazepam increased ESS scores in patients with PD. The results showed that ESS scores significantly increased after treatment with 6 weeks of 2 mg clonazepam (from 3.8 ± 1.2 to 7.3 ± 2.2; *p* = 0.0002) and were slightly increased after treatment with 6 weeks of 3 mg melatonin (from 4.1 ± 1.4 to 4.7± 1.4; *p* = 0.06; Litvinenko et al., 2012).

Delgado et al.'s study used 25 mg of melatonin to assess its efficacy on daytime sleepiness in patients with PD. Melatonin was taken at noon and 30 min before bedtime for 3 months. The results showed that the use of high-dose melatonin failed to reduce the presence of excessive daytime sleepiness (Delgado-Lara et al., 2020). Meanwhile, similar results were observed in that ESS scores after treatment with 4 weeks of 2 mg PRM were not different from baseline (Ahn et al., 2020).

In another study, Dowling et al. (2005) chose to compare 5 and 50 mg doses of melatonin vs. placebo over 2 weeks of administration. The results showed that compared with placebo, neither dose of melatonin significantly improved the ESS scores of patients with PD. Dowling et al. also used the general sleep disorder scale (GSDS) to assess the sleep of patients with PD and found that both daytime sleepiness and sleep quantity were significantly improved with 5 mg melatonin compared to either 50 mg or placebo (Dowling et al., 2005). The difference was statistically significant (*P* < 0.05).

### Effect of Melatonin on PD-RBD

Two studies (Ahn et al., 2020; Gilat et al., 2020) reported the effect of melatonin on RBD in patients with PD. In a 12-week, randomized, double-blind, placebo-controlled study, the weekly CIRUS-RBD Questionnaire (wCIRUS-RBDQ), was used to assess the efficacy of melatonin on RBD in patients with PD; the results showed that the number of RBD events after treatment with 8 weeks of 4 mg PRM was not reduced between groups (3.4 events/week with melatonin vs. 3.6 with placebo; absolute difference: 0.2; 95% confidence interval [CI] = −3.2 to 3.6; *P* = 0.92) (Gilat et al., 2020). The results showed that the number of nights in which a dream enactment event occurred during RBD events was not significantly different between groups (*P* = 0.56). In another randomized, double-blind, placebo-controlled, multicenter trial, Ahn et al. used the RBD screening questionnaire (RBDSQ) to investigate the efficacy of PRM in patients with PD. Coincidentally, the study of Ahn et al. also showed that the RBDSQ scores after treatment with 4 weeks of 2 mg PRM did not differ from baseline in either group (Ahn et al., 2020). Results of the qualitative analysis are shown in Table 2.

**Table 2:** Effect of melatonin vs. placebo on sleep disorders in Parkinson's disease.

**References**	**Sample size**	**Outcome measures**	***p*-value**	**Conclusion**
Daneshvar Kakhaki et al. (2020)	51	PSQI scale	*P* = 0.02 (PSQI)	Melatonin supplementation significantly reduced the PSQI scores compared with the placebo.
Ahn et al. (2020)	34	PSQI scale RBDQ ESS scale	*p* = 0.049 (PSQI) *p* = 0.716 (RBDQ)	PSQI score was improved in the melatonin group compared with baseline and the placebo group. Melatonin was an effective and safe treatment for PD patientspatients with PD with poor sleep quality. Post-treatment RBDSQ and ESS scores did not differ from baseline in either group.
Dowling et al. (2005)	40	ESS scale GSDS scale	Not reported (ESS) *P* < 0.05 (GSDS)	Daytime sleepiness as measured daily by the ESS did not improve during the two treatment periods; however, we did see a statistically significant improvement in weekly measure of the GSDS daytime sleepiness subscale scores for the 5 mg treatment period compared to placebo and 50 mg.
Gilat et al. (2020)	30	RBDQ	*P* = 0.92 (RBDQ)	No reduction in RBD was found between groups.
Medeiros et al. (2007)	18	PSQI scale ESS scale PSG	*p* = 0.03 (PSQI) *p* = 0.84 (ESS) *p* = 0.09 (total sleep time)	Melatonin significantly improved subjective quality of sleep. Daytime sleepiness is not affected by melatonin administration despite improved subjective sleep quality. No significant difference was observed in polysomnographic measures. A trend of improvement of total sleep time was observed in the melatonin-treated group.
Litvinenko et al. (2012)	38	ESS scale PSG	Not reported (ESS) *P* < 0.05 (PSG)	The ESS scores were significantly increased in the clonazepam group (from 3.8 ± 1.2 to 7.3 ± 2.2; *p* = 0.0002) and were slightly increased after treating with 6 weeks of 3 mg melatonin in the melatonin group (from 4.1 ± 1.4 to 4.7 ± 1.4; *p* = 0.06). Changes in total point scores on the PSG at the end of week 6, as compared with the beginning of the trial, were in favor of the group treated with melatonin, with significant changes in the LS (*p* = 0.004), total sleep time/time in bed (TST/TIB) (*p* = 0.001) sections.
Delgado-Lara et al. (2020)	26	ESS scale	*p* = 0.55 (ESS)	The presence of abnormal daytime and nighttime sleepiness continued after taking melatonin.

*PSQI, Pittsburgh sleep quality index; ESS, Epworth Sleepiness Scale; RBDQ, RBD questionnaire; PSG, Polysomnography; GSDS, General Sleep Disturbance Scale*.

## Discussion

PD is the second most common progressive neurodegenerative disorder. Studies have shown that mitochondrial dysfunction, abnormal protein handling, neuroinflammation, and oxidative stress play a key role in PD pathogenesis (Wood-Kaczmar et al., 2006; Michel et al., 2016). Mack et al. found that melatonin has neuroprotective, anti-inflammatory, and antioxidant properties (Mack et al., 2016). However, a series of studies have shown that the production of melatonin is reduced and the expression of melatoninergic receptors MT1 and MT2 is decreased in the substantia nigra pars compacta (Fertl et al., 1991, 1993; Bordet et al., 2003; Adi et al., 2010; Videnovic et al., 2014). Therefore, an increasing number of scholars are studying the efficacy of exogenous melatonin in patients with PD (Delucca et al., 2018; Ahn et al., 2020; Daneshvar Kakhaki et al., 2020; Gilat et al., 2020; Batla et al., 2021). Sleep disorders are one of the most common non-motor symptoms of patients with PD. However, the effect of melatonin on sleep disorders in patients with PD is unclear. Therefore, we performed a meta-analysis of RCTs and systematic review to comprehensively investigate the efficacy of melatonin in the treatment of sleep disorders in patients with PD.

### Melatonin on Insomnia

Insomnia is one of the most common sleep disorders in patients with PD. Insomnia is defined as complaints of difficulty initiating sleep and/or difficulty maintaining sleep and/or early morning awakenings, according to the International Classification of Sleep Disorders Third Edition criteria (Sateia, 2014). Clinical interviews are the gold standard to determine the diagnosis of insomnia. The interview gathers information on the type of insomnia complaint, etiology, duration, and repercussions (Wallace et al., 2020). In addition to the clinical interview, some psychometrically validated scales can also be used to evaluate the subjective sleep of patients with PD, such as the PSQI scale, the Insomnia Severity Index (ISI), the Scales for Outcome in Parkinson's Disease-Sleep (SCOPA-S) and the Parkinson's Disease Sleep Scale (PDSS) (Buysse et al., 1989; Chaudhuri et al., 2002; Marinus et al., 2003; Trenkwalder et al., 2011; Suzuki et al., 2015; Wallace et al., 2020).

The PSQI scale is an effective tool for measuring subjective sleep quality. In recent expert consensus recommendations, the PSQI scale has been recommended as the main measure for global sleep and insomnia symptoms (Buysse et al., 2006). In our study, we used the PSQI scale to evaluate the subjective sleep quality of patients with PD. The scale is a self-rated questionnaire that is used to evaluate sleep quality and sleep disorders over a 1-month time interval (Buysse et al., 1989). Nineteen separate items produced seven component scores, including subjective sleep quality, sleep latency, sleep duration, habitual sleep efficiency, sleep disturbances, use of sleeping medication, and daytime dysfunction (Buysse et al., 1989). The score of each component can take the value of 0, 1, 2, or 3. The sum of the scores of the seven components is used to calculate the overall sleep quality score, which ranges from 0 to 21 (Snyder et al., 2018). The higher the score is, the worse the quality of sleep. Our meta-analysis and systematic review is the first study to evaluate the effects of melatonin on the subjective and objective sleep quality of patients with PD. Many studies have shown that melatonin can improve the subjective sleep quality of PD (Zhang et al., 2016; Fatemeh et al., 2021). A meta-analysis showed that melatonin could improve subjective sleep quality in neurodegenerative disorder patients (Zhang et al., 2016). Our study also showed that melatonin could significantly improve the subjective sleep quality of patients with PD (*P* = 0.001), which is consistent with previous results. To avoid false-positive results caused by random errors, we used TSA software to perform trial sequential analysis. According to the TSA results, we concluded that melatonin can improve the PSQI scores in patients with PD.

PSG is an objective sleep measure. In our study, we used PSG to objectively evaluate the sleep of patients with PD. However, PSG is not indicated in the routine assessment of adults with insomnia, and it may be necessary when comorbid sleep disorders are suspected (Wallace et al., 2020). Therefore, only a few articles use PSG to monitor objective sleep in patients with PD. Two studies objectively evaluated the efficacy of melatonin on the objective sleep quality of patients with PD using PSG. In our study, the results indicate that melatonin had a significant effect on total sleep time/time in bed (TST/TIB) and latent sleep (LS) sections of the PSG. Meanwhile, we also observed a trend of improvement in total sleep time (TST) in another study (Medeiros et al., 2007). Actigraphy is another measure of evaluating objective sleep. In Dowling et al.'s study, the authors found a statistically significant improvement in actigraphically measured total sleep time with 50 mg melatonin compared to 5 mg melatonin or placebo (Dowling et al., 2005).

### Melatonin on Excessive Daytime Sleepiness

Excessive daytime sleepiness is one of the most common non-motor symptoms of PD. It has been reported that most PD patients suffer from excessive daytime sleepiness (Ondo et al., 2001; Tracik and Ebersbach, 2001; Ghorayeb et al., 2007; Poryazova et al., 2010), which seriously impairs the quality of life of patients with PD (Schrempf et al., 2014). Because of sleep disorders, especially sudden sleep attacks, many activities are dangerous for patients with PD, such as driving a car or operating a machine.

The ESS scale is a widely used tool, and it has been verified as a measure of sleepiness (Walker et al., 2020). The ESS scale is an eight-item self-applicable instrument that is used to assess the propensity to fall asleep in eight mostly monotonous situations. A total score of <10 is rated as normal, a score of 10 to 12 indicates marginal sleepiness and a score higher than 12 indicates excessive sleepiness (Sandoval-Rincón et al., 2013). In our included studies, ESS scores after treatment with melatonin were not improved compared with those of the control group, regardless of the dose of melatonin and treatment duration. However, it has been questioned whether ESS was used as an evaluative instrument to measure change over time or responsiveness to treatment. Dowling's study showed that daytime sleepiness and sleep quantity significantly improved in weekly measures of the General Sleep Disturbance Scale (GSDS) daytime sleepiness subscale scores for the 5 mg melatonin treatment compared to placebo and 50 mg melatonin (Dowling et al., 2005). However, these results were not consistent in the ESS scores. The GSDS and ESS assess sleepiness across different periods and measure slightly different aspects of sleepiness, which may be explain the findings. In our study, melatonin did not improve excessive daytime sleepiness assessed with the ESS scale in patients with PD compared with the control group. However, we observed that melatonin improved GSDS daytime sleepiness subscale scores after treatment with 5 mg melatonin. Therefore, more studies that use different scales to evaluate the effect of melatonin on daytime sleepiness in patients with PD will be needed. The multiple sleep latency test (MSLT) is an objective assessment of daytime function in patients with chronic insomnia. The MSLT can also be used in research (Stepanski et al., 1989; Zhang and Zhao, 2007). Research has shown that MSLT assessment has proven useful in evaluating a broad range of patients with excessive daytime sleepiness (Roth and Ancoli-Israel, 1999).

### Melatonin on PD-RBD

Rapid eye movement (REM) sleep behavior disorder (RBD) is a parasomnia characterized by abnormal behaviors and loss of muscle atonia, such as vocalizations, jerks and motor behaviors during REM sleep; it is often related to REM-related dream content (Schenck and Mahowald, 2002; Zhang et al., 2020). RBD can lead to dream enactment behaviors that are often injurious to patients and their partners (Haba-Rubio et al., 2018). The prevalence of RBD is approximately 1% in adults but 20–50% in people with PD (Dauvilliers et al., 2018; Haba-Rubio et al., 2018; Hogl et al., 2018). Studies have shown that in evolving neurodegenerative disorders, idiopathic RBD (iRBD) is considered to be the first step (Iranzo et al., 2006, 2013). In a longitudinal study of 29 patients, 38% of patients with iRBD developed PD, MSA or DLB within 4 years of evaluation, and 81% developed neurodegenerative diseases after 20 years (Schenck et al., 2013). The diagnosis of RBD requires the clinical history of dream enactment behaviors (DEBs) or REM sleep-related behaviors recorded by PSG, along with REM sleep without atonia (RSWA) (St Louis and Boeve, 2017). The key to diagnosing RBD is the witnessing of dream enactment by a bed partner (Chiaro et al., 2018). Some questionnaires, such as the RBD Screening Questionnaire (RBDSQ), the REM Sleep Behavior Questionnaires-Hong-Kong (RBD-HK), the Mayo Sleep Questionnaire (MSQ), and the Innsbruck RBD Inventory, can also be used in the diagnosis of RBD (Jin et al., 2017). However, the pathogenesis of RBD is still not clear. It is speculated that the degeneration or dysfunction of the brain stem circuits controlling rapid eye movement sleep paralysis is the root cause of RBD (McKenna and Peever, 2017).

Studies have shown that melatonin has a much safer profile than clonazepam, with no reports of dependence and fewer and milder side effects (Aurora et al., 2010; Dauvilliers et al., 2018). Therefore, melatonin is considered a preferable treatment for RBD in older patients and neurodegenerative disorder patients (Kunz and Bes, 1997, 1999; Aurora et al., 2010; Kunz and Mahlberg, 2010; Dauvilliers et al., 2018). However, a few studies have investigated the efficacy of melatonin on PD-RBD. In a two-part, double-blind, placebo-controlled trial, melatonin was effective in RBD patients (Kunz and Mahlberg, 2010). Eight consecutive outpatients were included in the study, but only one was suffering from PD. Gilat recruited 30 PD participants who were confirmed to have RBD with video PSG. The 30 PD patients with RBD were randomized to 4 mg of prolonged-release melatonin or placebo, and the aggregate of RBD incidents averaged was used as the primary outcome (Gilat et al., 2020). Gilat's results showed that melatonin did not significantly improve the clinical symptoms of RBD compared to placebo (Gilat et al., 2020). In another randomized controlled study (Ahn et al., 2020), the same result was observed. Based on the available evidence, we cannot confirm that melatonin is effective for PD-RBD. Therefore, larger, multicentered RCTs are still needed to assess the effect of melatonin on PD-RBD.

### Melatonin on Other Sleep Disorders

Other sleep disorders are also often present in patients with PD, such as restless leg syndrome, periodic leg movements during sleep, and obstructive sleep apnea (Iranzo, 2016). However, no recent study has evaluated the efficacy of melatonin on these sleep disorders.

### Adverse Reactions

Melatonin is well-tolerated. One study showed that no significant side effects were reported by participants taking 20–100 mg/day melatonin orally, and no important alterations to any physiological or biochemical measures were seen (Galley et al., 2014). In previous studies, the safety of melatonin was established in patients with PD (Dowling et al., 2005; Medeiros et al., 2007). In our study, no serious adverse events occurred when the dose of melatonin was as high as 50 mg/day in patients with PD. This conclusion is consistent with previous studies.

Headache and daytime sleepiness were frequently reported and were the most common adverse reactions during melatonin treatment. In Ahn and Medeiros's studies (Medeiros et al., 2007; Ahn et al., 2020), no participants experienced adverse events. In Daneshvar's study, grade 1 side effects were reported in two PD participants in the melatonin group, including headache (*n* = 1) and daytime sleepiness (*n* = 1) (Daneshvar Kakhaki et al., 2020). In Dowling's study, one participant complained of feeling tired in the morning during treatment with 50 mg melatonin but insisted on completing the treatment (Dowling et al., 2005). In Gilat's study, because one participant felt light-headedness and morning sleepiness, the dosage of melatonin was reduced from 4 to 2 mg after 3 weeks (Gilat et al., 2020). In two studies, adverse events were reported, including headaches, fatigue, light-headedness, daytime sleepiness, dizziness, nausea, or gastrointestinal problems (Delgado-Lara et al., 2020; Gilat et al., 2020). However, these adverse events were mild and did not require further medical attention.

### Strengths and Limitations

Our study has several major strengths: (1) This study is the first meta-analysis and systematic review that investigated the efficacy of melatonin on sleep disorders in patients with PD. (2) Previous systematic reviews and meta-analyses only discussed the effect of melatonin on subjective sleep quality. Our study is the first to evaluate the effects of melatonin on the subjective and objective sleep quality of patients with PD. However, due to data sparseness and repeated testing of the accumulated data, the accumulated meta-analysis has the risk of producing random errors. In our study, TSA was used to test whether the conclusions of the meta-analysis were sufficient.

There are several potential limitations in our study: (1) The dosage and duration of melatonin used in each included study were different. Because measures to assess the efficacy of melatonin on sleep disorders in patients with PD were different, we could not perform subgroup analysis. (2) There were only seven RCTs that met the inclusion criteria, and the sample size of each study was small.

## Conclusion

In conclusion, the combined data from RCT studies showed that melatonin could significantly improve the subjective and objective sleep quality of patients with PD with good safety and tolerability. Melatonin could be considered an effective treatment for insomnia in patients with PD, which provides evidence for clinicians to select drugs for sleep disorders in patients with PD and provides a basis for experts to formulate guidelines. However, we also found that there was no significant improvement in PD-RBD and excessive daytime sleepiness assessed by the ESS scale. Therefore, a larger, multicentered RCT will still be needed to assess the efficacy of melatonin on sleep disorders in patients with PD.

## Data Availability Statement

The original contributions presented in the study are included in the article/Supplementary Material, further inquiries can be directed to the corresponding author/s.

## Author Contributions

JY and HM designed the study. WS, MJ, and YZ carried out the literature searches, study selection, and data extraction. HM and WS assessed the quality of studies, conducted the trial sequential analysis, contributed to the analysis, and interpreted the data. HM wrote the manuscript. JY revised the manuscript. All authors contributed to the writing of this manuscript.

## Funding

This work was supported by the Project of Henan Province Science and Technology (212102310216), the Key Projects of Medical Science and Technology in Henan Province (SBGJ202002099) and Medical Science and Technology Research in Henan Province (LHGJ20190560).

## Conflict of Interest

The authors declare that the research was conducted in the absence of any commercial or financial relationships that could be construed as a potential conflict of interest.

## Publisher's Note

All claims expressed in this article are solely those of the authors and do not necessarily represent those of their affiliated organizations, or those of the publisher, the editors and the reviewers. Any product that may be evaluated in this article, or claim that may be made by its manufacturer, is not guaranteed or endorsed by the publisher.
