# Supplementary material for: Melatonin Treatment for Sleep Disorders in Parkinson's Disease: A Meta-Analysis and Systematic Review
Source: Front Aging Neurosci. 2022 Feb 4;14:784314. doi: 10.3389/fnagi.2022.784314 (PMC8855052; doi:10.3389/fnagi.2022.784314)
Supplement: Supplementary file 1 [file Data_Sheet_1.docx]

**Supplementary Material 1 :** **The Search Strategy on PubMed**

- **#**1 **(**Parkinson Disease [mh] ) OR Idiopathic Parkinson's Disease OR Lewy Body Parkinson's Disease OR Parkinson's Disease, Idiopathic OR Parkinson's Disease, Lewy Body OR Parkinson Disease, Idiopathic OR Parkinson's Disease OR Idiopathic Parkinson Disease OR Lewy Body Parkinson Disease OR Primary Parkinsonism OR Parkinsonism, Primary OR PD OR Paralysis Agitans
- #2 **(**Melatonin [mh] ) OR melatonin

#3 Daytime Somnolence OR Daytime Somnolences OR Somnolence, Daytime OR Daytime Sleepiness OR Daytime Sleepinesses OR Sleepiness, Daytime OR Excessive Daytime Sleepiness OR Daytime Sleepiness, Excessive OR Excessive Daytime Sleepinesses OR Sleepiness, Excessive Daytime OR Insomnia OR Behavior Disorder, REM OR Behavior Disorders, REM OR REM Behavior Disorders OR REM Behavior Disorder OR Behavior Disorder, Rapid Eye Movement Sleep OR Rapid Eye Movement Sleep Behavior Disorder OR Restless Legs OR Restless Leg Syndrome OR Syndrome, Restless Leg OR Apneas, Obstructive Sleep OR Obstructive Sleep Apneas OR Sleep Apneas, Obstructive OR Obstructive Sleep Apnea Syndrome OR Obstructive Sleep Apnea OR OSAHS OR Syndrome, Sleep Apnea, Obstructive OR Sleep Apnea Syndrome, Obstructive OR Apnea, Obstructive Sleep OR Sleep Apnea OR Hypopnea Syndrome OR Syndrome, Obstructive Sleep Apnea OR Upper Airway OR Resistance Sleep Apnea Syndrome OR Syndrome, Upper Airway Resistance, Sleep Apnea OR periodic leg movements during sleep

- #4 **#**1 AND #2 AND #3
- #5 randomized controlled trial [pt]
- #6 controlled clinical trial [pt]
- #7 randomized [ti/ab]
- #8 placeb [ti/ab]
- #9 randomly [ti/ab]
- #10 tria [ti/ab]
- #11 groups [ti/ab]
- #12 #5 OR #6 OR #7 OR #8 OR #9 OR #10 OR #11
- #13 #4 AND #12
